# Thermal stability of 2DEG at amorphous LaAlO_3_/crystalline SrTiO_3_ heterointerfaces

**DOI:** 10.1186/s40580-016-0067-9

**Published:** 2016-04-01

**Authors:** Seon Young Moon, Cheon Woo Moon, Hye Jung Chang, Taemin Kim, Chong-Yun Kang, Heon-Jin Choi, Jin-Sang Kim, Seung-Hyub Baek, Ho Won Jang

**Affiliations:** 1 Center for Electronic Materials, Korea Institute of Science and Technology, Seoul, 136-791 Republic of Korea; 2 Department of Materials Science and Engineering, Yonsei University, Seoul, 120-749 Republic of Korea; 3 Department of Materials Science and Engineering, Research Institute of Advanced Materials, Seoul National University, Seoul, 151-744 Republic of Korea; 4 Department of Nanomaterials Science and Technology, Korea University of Science and Technology, Daejeon, 305-350 Republic of Korea; 5 KU-KIST Graduate School of Converging Science and Technology, Korea University, Seoul, 136-701 Republic of Korea

**Keywords:** 2-Dimensional electron gas, Oxide, Interface, Thermal stability

## Abstract

At present, the generation of heterostructures with two dimensional electron gas (2DEG) in amorphous LaAlO_3_ (a-LAO)/SrTiO_3_ (STO) has been achieved. Herein, we analysed thermal stability of 2DEG at a-LAO/STO interfaces in comparison with 2DEG at crystalline LaAlO_3_ (c-LAO)/STO interfaces. To create 2DEG at LAO/STO interface, regardless of growing temperature from 25 to 700 °C, we found that environment with oxygen deficient during the deposition of LAO overlayer is essentially required. That indicates that the oxygen-poor condition in the system is more essential than the crystalline nature of LAO layer. 2DEG at a-LAO/STO interface is depleted upon ex situ annealing at 300 °C under 300 Torr of oxygen pressure, while that in c-LAO/STO interface is still maintained. Our result suggests that the LAO overlayer crystallinity critically affects the thermal-annealing-induced depletion of 2DEG at a-LAO/STO interface rather than the generation of 2DEG. We clearly provide that amorphous TiO_x_ can efficiently prevent the thermal degradation of 2DEG at the a-LAO/STO interface, which gives a cornerstone for achieving thermal-stable 2DEG at a-LAO/STO interface.

## Background

Oxide-based complex materials having strongly correlated electrons play a crucial role in a wide variety of physical phenomena and have potential for applications in exotic fields such as charge-ordered insulators, high temperature superconductivity, unconventional ferroelectricity and double exchange ferromagnets [1–3]. With improvements in atomic-scale synthesis [4, 5], heterointerfaces between complex oxides are emerging as highly interesting electronic systems owing to their unique properties [6–9]. Such interfaces can generate electron systems that are not found in nature in bulk. A recent prominent finding is that a high mobility two-dimensional electron gas (2DEG) can be generated at the interface between two insulating oxides, LaAlO_3_ (LAO) and SrTiO_3_ (STO), and the formation of 2DEG is confined within a layer of ~1 nm from the LaO/TiO_2_ interface [10–16]. At this polar interface both superconductivity and ferromagnetism can coexist as a result of electronic phase separation [17–20]. The interfacial conductivity shows a number of intriguing properties such as on/off switching with external electric fields [12, 21], nanoscale electronic devices [21, 22], and tunable conductivity controlled using the strong surface-interface interaction by the surface adsorbates [23, 24].

Recently, the formation of 2DEG can be found in various STO-based heterointerfaces which are different from the conventional (001) LAO/STO systems in terms of the crystal orientation and the overlayer materials [25–30]. Metallic interfaces can be realized in epitaxial LAO layers on (110) and (111) STO substrates which have no interface polar discontinuity surfaces and exhibits 2DEG transport with mobilities similar to those of (001) STO [31]. The 2DEG can be constructed at with various amorphous overlayers of LAO, STO and yttria-stabilized zirconia (YSZ) [32]. The formation of 2DEG was attributed to oxygen vacancies constrained near the interface, suggesting that the redox reactions on the surface of STO substrates play an important role [25]. Remarkably, the 2DEG at spinel γ-Al_2_O_3_/perovskite STO interface showed an astonishingly high mobility of approximately 1.4 × 10^5^ cm^2^ V^−1^ s^−1^ [26]. The mobilities are much higher than those (~30,000 cm^2^ V^−1^ s^−1^) of the Nb-doped STO single crystal and the La-doped STO film [33–36]. These studies are expected to trigger intensive studies on high mobility complex oxide interfaces as conducted for III–V or Si–SiGe semiconductors [37–39]. Meanwhile, there is little discussion about the reliability of the 2DEG oxide interfaces. A deep understanding of the physical parameters governing the stability of 2DEG in oxide-based complex materials is crucial for real device applications. For this reason, it is important to investigate thermal stability of 2DEG in STO-based heterointerfaces for both amorphous and crystalline LAO/STO heterointerfaces, which has not been done yet.

In this study, we report both the formation and thermal-annealing-induced annihilation of 2DEG at amorphous LAO/STO (a-LAO/STO) heterointerface. The experimental results for a-LAO/STO system are compared with those for the most standardized crystalline LAO/STO (c-LAO/STO) system. Our electrical measurements reveal that the metallic conductivity at a-LAO/STO heterointerfaces is obtained in oxygen-deficient growth conditions and that there is a critical thickness of the a-LAO overlayer for the 2DEG formation, which are very similarly observed in c-LAO/STO heterointerfaces. However, a-LAO/STO shows much pronounced degradation in 2DEG conductivity compared with c-LAO/STO with increasing ex situ annealing temperature, demonstrating that the crystallinity of the overlayer has little impact on the 2DEG formation but significantly affect the thermal stability of 2DEG in the STO-based heterointerfaces.

## Experimental details

All LAO overlayers were grown on TiO_2_-terminated STO substrates by pulsed laser deposition (PLD) in an oxygen atmosphere. Two types of LAO overlayers were grown. In the first, we deposited a LAO overlayer at room temperature, resulting in an amorphous LAO (a-LAO). Structural characterizations of the heterostructures were performed using a scanning transmission electron microscope (STEM, Titan, FEI) operated at 300 kV. In the second, we deposited a LAO overlayer at 700 °C, resulting in a crystalline LAO (c-LAO). Both types of heterostructures were grown with various LAO overlayer thicknesses varying from 0 to 12 nm. Both types were grown at the oxygen pressure of 1 mTorr. After the deposition of c-LAO overlayers at 700 °C, the samples were cooled down to room temperature maintaining the oxygen pressure at 1 mTorr. The laser energy density of 1.5 J cm^−2^ and the repetition rate of 2 Hz were used. The distance from the target surface to the sample was 50 mm. In addition, we deposited various amorphous capping layer such as TiO_2_, SnO_2_, SiO_2_, and Al_2_O_3_ on the a-LAO/STO heterostructure at room temperature using RF magnetron sputtering and PLD. The thickness of all amorphous capping layers is 100 nm. The interfacial conductivity at room temperature was evaluated using current–voltage (*I*–*V*) measurements which had been carried out using indium ohmic contacts on the diagonal corners of 5 mm × 5 mm samples. The sheet resistance and carrier concentration were measured by a Hall measurement system using the van der Pauw configuration in the temperature range from 300 K down to 10 K. And the electron mobility was calculated from the relationship between sheet resistance and carrier concentration. To test the stability of the a-LAO/STO and c-LAO/STO heterostructures, they were ex situ annealed at temperatures ranging from 100 to 700 °C for 1 h under the oxygen pressure of 300 Torr.

## Results and discussion

**Fig. 1:**
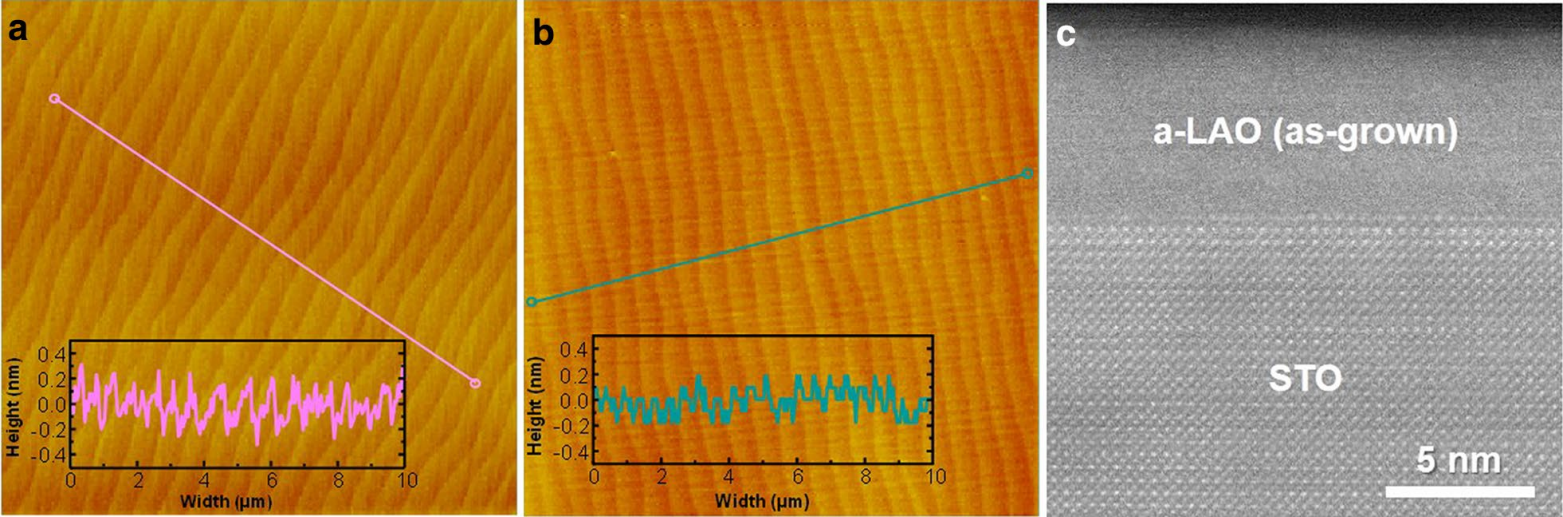
AFM images of 5-nm-thick a-LAO (**a**) and c-LAO (**b**) films grown on STO substrate. The *insets* are surface profiles of the a-LAO/STO (rms roughness of 0.25 nm) and c-LAO/STO (rms roughness of 0.26 nm) heterostructures. The step heights of LAO/STO heterostructures (~0.4 nm) correspond to one unit-cell of LAO. **c** Cross-sectional high-angle annular dark field STEM images for an as-grown a-LAO/STO heterostructure

Figure 1a, b shows AFM images of a-LAO/STO (5-nm-thick LAO overlayer grown at room temperature under $P_{{{\text{O}}_{2} }}$ = 1 mTorr) and c-LAO/STO (5-nm-thick LAO overlayer grown at 700 °C under $P_{{{\text{O}}_{2} }}$ = 1 mTorr) heterostructures with their representative height profiles. The a-LAO/STO (Fig. 1a) and c-LAO/STO (Fig. 1b) surfaces consist of regular steps and terraces structures that are inherent in the TiO_2_-terminated substrates. The height profiles of the a-LAO/STO and c-LAO/STO surfaces indicate that the step height is about 0.4 nm which corresponds to one unit-cell of LAO. The root mean square (rms) roughness of the a-LAO/STO surface (0.26 nm) was very similar to that of the c-LAO/STO surface (0.25 nm). It indicates that the a-LAO overlayer as well as c-LAO layer has very smooth surface and is a uniform thickness over the area, replicating the atomically flat step-terrace surface of the substrate. Figure 1c displays the cross-sectional high-angle annular dark field STEM images of an as-grown a-LAO/STO heterostructure. For the as-grown sample, no periodic lattices at the LAO overlayer reveal that the LAO layer is amorphous.

**Fig. 2:**
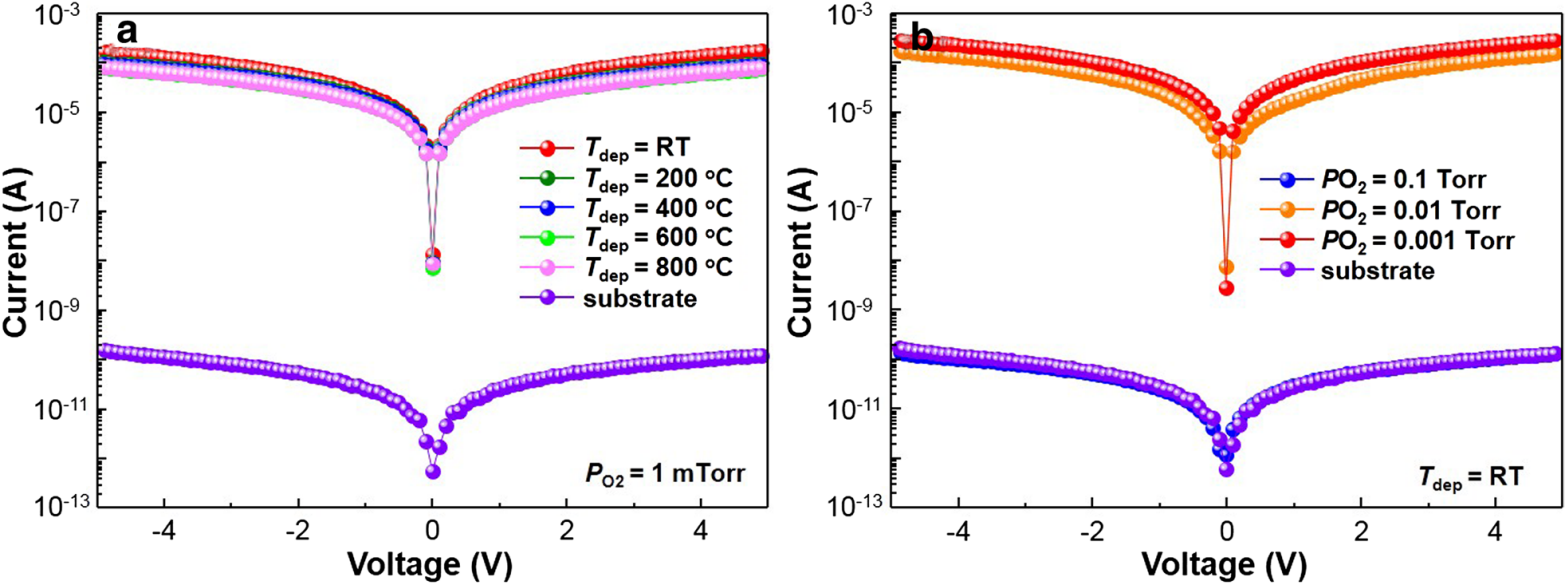
*I*–*V* characteristics of LAO/STO heterostructures grown at **a** different deposition temperatures (*T*_dep_) and **b** different oxygen partial pressures ($P_{{{\text{O}}_{2} }}$). The thickness of the LAO overlayers was kept to be 5 nm. For comparison, an *I*–*V* characteristic of bare STO substrate is presented

*I*–*V* characteristics of LAO/STO heterostructures grown at different temperatures are shown Fig. 2a. The oxygen pressure ($P_{{{\text{O}}_{2} }}$) and the thickness of the LAO overlayers were fixed at 1 mTorr and 5 nm, respectively. All the LAO/STO heterostructures are conducting regardless of the growth temperature. Our previous reports [22, 23, 34] showed that 2DEG forms at LAO/STO heterostructures grown at 500–700 °C under $P_{{{\text{O}}_{2} }}$ = 1 mTorr, indicating that 2DEG forms even with the a-LAO overlayer and the crystallinity of the LAO overlayer is rarely related to the interfacial conductivity. The conductivity of a-LAO/STO heterostructures is strongly dependent on the $P_{{{\text{O}}_{2} }}$ during film growth (Fig. 2b), which is consistent with the previous reports [10, 24]. The a-LAO/STO heterostructures grown at $P_{{{\text{O}}_{2} }}$ = 100 mTorr is insulating same as the STO substrate. Upon decreasing the $P_{{{\text{O}}_{2} }}$ below 10 mTorr, the a-LAO/STO heterointerfaces are conducting. These results suggest that oxygen deficient atmosphere rather than the crystallinity of the LAO overlayer is a key factor to form 2DEG at LAO/STO heterointerfaces. The sheet conductance of a-LAO/STO and c-LAO/STO heterostructures were measured as a function of the thickness of LAO overlayer at room temperature. Insulator-to-metal transitions at specific thicknesses of LAO overlayer were observed regardless of the crystallinity of LAO overlayer. When the c-LAO overlayer reaches 1.6 nm thickness, the sheet conductance rose by 5-orders of magnitude. After the abrupt change, the conductance was constant while the thickness of LAO overlayer increases. For a-LAO/STO heterostructures, the critical thickness of 2DEG formation was found to be 2.6 nm. The sheet conductance of 2DEG at a-LAO/STO interfaces was analogous to that at c-LAO/STO interfaces, meaning that 2DEG conductivity is irrelevant to the crystallinity of LAO overlayer. This indicates that carriers are confined at LAO/STO interfaces as 2DEG, consistent with previous reports [12, 40, 41].

**Fig. 3:**
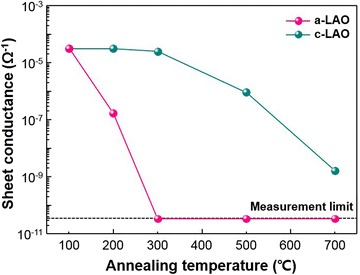
Sheet conductance of a-LAO/STO and c-LAO/STO heterostructures as a function of annealing temperature

The thermal stability of 2DEG in a-LAO/STO and c-LAO/STO heterostructures was evaluated by ex situ annealing at various temperatures under oxygen ambient (300 Torr) for 1 h. a-LAO/STO and c-LAO/STO heterostructures exhibited similar *I*–*V* curves after annealing at 100 °C. But dissimilarity was found as the annealing temperature increased. For the a-LAO/STO heterostructure, the current at 5 V was reduced from 156 µA to 841 nA after annealing at 200 °C. Annealing at 300 °C made the a-LAO/STO heterostructure insulating like the bare STO substrate. For the c-LAO/STO heterostructure, the degradation of 2DEG conductivity by annealing at 300 °C was negligible. To clearly juxtapose thermal degradation trait of the a-LAO/STO and c-LAO/STO heterostructures, the sheet conductances of the various heterostructures as a function of annealing temperature are plotted in Fig. 3. The a-LAO/STO heterostructure show relatively very rapid thermal degradation while the c-LAO/STO one still exhibits a finite interfacial conductance after annealing at 700 °C. Chen et al. [32] discussed the thermal unstability of 2DEG at a-LAO/STO, stating that the conductivity in amorphous STO-based heterostructures can be removed by annealing in 0.5–1 bar pure O_2_ at 150–300 °C.

**Fig. 4:**
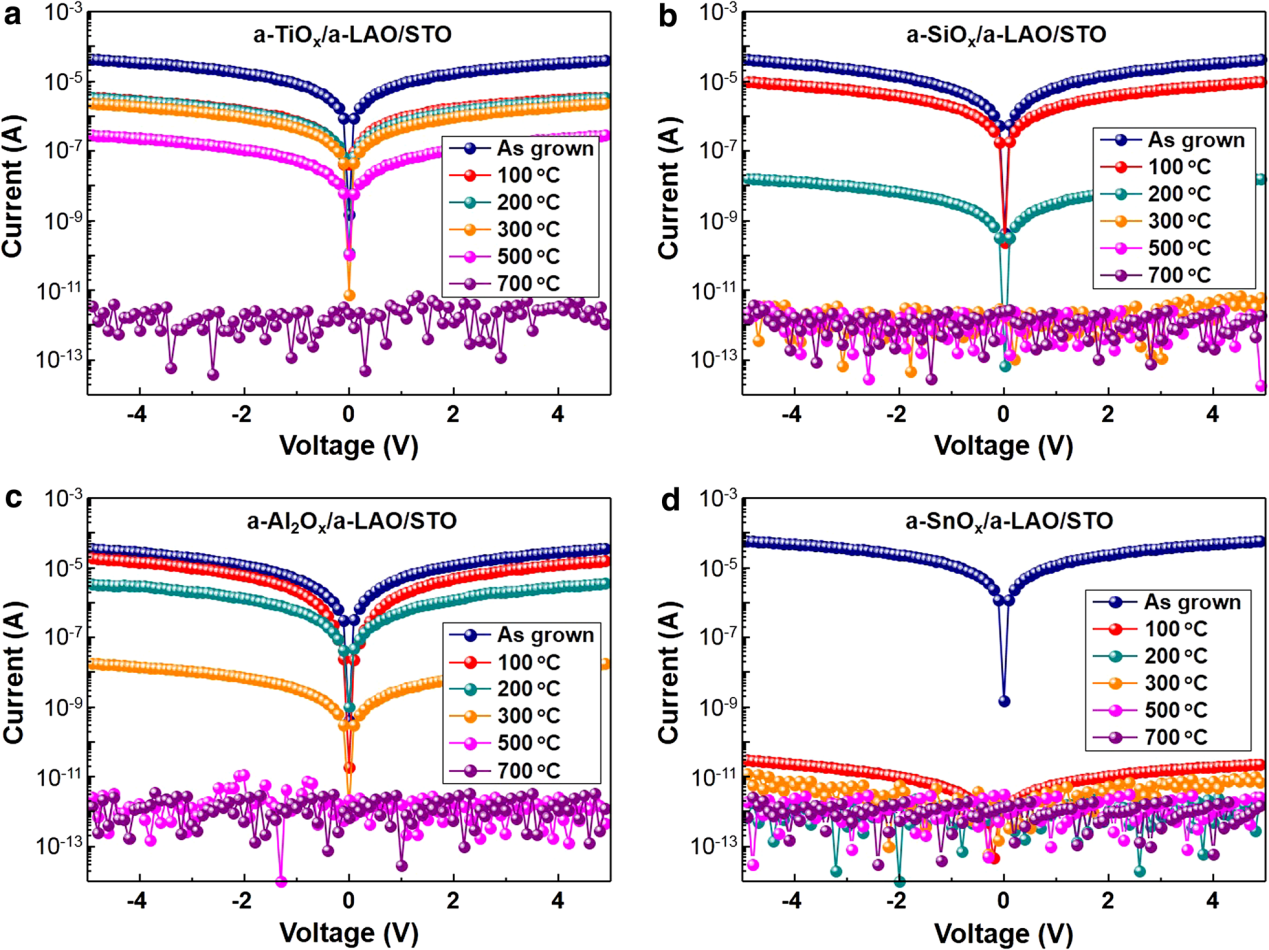
*I*–*V* characteristics of **a** a-TiO_x_/a-LAO/STO, **b** a-SiO_x_/a-LAO/STO, **c** a-AlO_x_/a-LAO/STO and **d** a-SnO_x_/a-LAO/STO heterostructures after annealing at various temperatures under the oxygen pressure of 300 Torr. The thickness of the MeO_x_ overlayers is 100 nm and the a-LAO layer is 5-nm-thick

In order to improve the thermal stability of 2DEG at a-LAO/STO interface, the a-LAO surface should be prohibited from being exposed to high oxygen pressure at elevated temperatures. To accomplish this, we have deposited amorphous 100-nm-thick TiO_x_, SiO_x_, Al_2_O_3_ and SnO_x_ layers on a-LAO (5 nm)/STO heterostructure as oxygen barrier layer using RF sputtering at room temperature. Figure 4 shows *I*–*V* characteristics of a-MeO_x_/LAO/STO heterostructures before and after annealing. It is clearly seen that the a-TiO_x_ layer effectively retards the rapid thermal degradation of 2DEG at the a-LAO/STO interface. After annealing at 300 °C, 2DEG conductance maintained persistently at the heterointerfaces with the TiO_x_ and AlO_x_ overlayers.

**Fig. 5:**
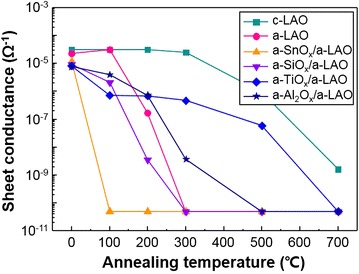
Sheet conductance change of a-LAO/STO under various capping overlayers

Figure 5 shows sheet conductance change of a-MeO_x_/LAO/STO heterostructures as a function of annealing temperature. The a-TiO_x_ overlayer prevents the rapid thermal degradation of 2DEG at the a-LAO/STO interface. 2DEG conductance did not maintain at the heterointerfaces after annealing at 300 °C with the SnO_x_ and SiO_x_ overlayers. That means those overlayers did not prevent the thermal degradation. Although the reason why improving the thermal stability of 2DEG at the a-LAO/STO interface using a-MeO_x_ overlayers is strongly material-dependent and yet illusive now, this result clearly shows that an additional overlayer on the a-LAO/STO heterostructure plays a role in enhancing the thermal stability of 2DEG at the heterointerface.

## Conclusion

In this work, we investigated the thermal stability of 2DEG at a-LAO/STO interface. 2DEG is formed under oxygen deficient growth condition and the crystallinity of LAO overlayer is rarely related to the 2DEG formation. The electrical properties of 2DEG at a-LAO/STO interface are analogous to those at c-LAO/STO interface. We found that the thermal stability of 2DEG upon annealing in high oxygen pressure is strongly dependent on the crystallinity of LAO overlayer. We suggested that preserving the surface oxygen vacancies is critical in achieving thermal stable 2DEG interface at elevated temperatures. It is demonstrated that an additional overlayer like a-TiO_x_ on a-LAO/STO heterostructure can enhance the thermal stability of 2DEG at the heterointerface.
